# Benefits of Being Teamed with a Service Dog for Individuals Living with Visible and Invisible Disabilities

**DOI:** 10.3390/healthcare11222987

**Published:** 2023-11-19

**Authors:** Joanne K. Singleton

**Affiliations:** Lienhard School of Nursing, College of Health Professions, Pace University, New York, NY 10038, USA; jsingleton@pace.edu

**Keywords:** disabilities, service dogs, benefits

## Abstract

Over 61 million people in the United States are living with disabilities. Less than one percent are teamed with service dogs. A service dog is a type of assistance dog specifically trained to perform a disability-related task(s) to assist the person and support their independence. Service dogs may also provide valuable secondary benefits. The aim of this survey research is to add empiric data on benefits of being teamed with a service dog. Two hundred and four individuals teamed with service dogs responded to demographic and survey questions that included the benefits they experience from being teamed with a service dog. Overwhelmingly, respondents agreed or strongly agreed to benefits of emotional connection (96%), community participation (97%), physical activity (96%), psychological wellbeing (98%), quality of life (97%), a reduction in prescribed medications (78%), and a decrease in paid or unpaid assistance hours (83%), which extend beyond their primary disability need. It is clear that many others may benefit from being teamed with a service dog. Greater evidence is needed to increase tangible support for those who desire to be teamed with a service dog and can meet the Americans with Disabilities requirements. Additionally, to support individuals with service dogs now and in the future, healthcare professionals need to be knowledgeable about and culturally competent in caring for patients teamed with service dogs. Service dogs, when indicated, may benefit individuals living with disabilities by meeting primary and secondary needs that support independence.

## 1. Introduction

Over 61 million Americans are living with visible and invisible disabilities [1]. A critical goal for healthcare providers in working with these individuals is to support their independence and ability to interact within society as fully as possible. One strategy that has been in practice for decades is for the person to be teamed with an assistance dog. Definitional clarity is essential for developing knowledge in the field of assistance dogs. In the United States (US), the Americans with Disabilities Act (ADA, 2023) broadly defines service dogs, while the definition by Assistance Dogs International (ADI) is more specific. ADI defines a service dog as a type of assistance dog extensively trained to work with a person who’s disability is not blindness or deafness [2,3]. While there are many similarities across service dogs, ADI recognizes differences in the specific needs of canine support for those with vision and hearing disabilities. Additionally, there has been little research to date that focuses on service dogs based on the ADI definition. Gaining knowledge about all individuals teamed with service dogs using the broad ADA definition is equally important and will be taken up and reported on in a future article. The focus of this research uses the ADI definition and includes individuals teamed with service dogs, other than for blindness or deafness.

The work of the service dog is to support the person by performing a specific disability-related task. Individuals in the United States who are teamed with a service dog have the right to be accompanied by their service dog anywhere the public is allowed as long as the service dog meets expected standards for their behavior in public. Not all individuals with disabilities may be suitable to be teamed with a service dog, as the person, known as the handler, (or their proxy when it is a child), must be in control of the service dog at all times [3].

The Americans with Disabilities Act (ADA) recognizes that a service dog may be trained by an accredited service dog organization and may also be self-trained by the individual with the disability for which the person often enlists support from a dog trainer [3]. National statistics on individually trained service dogs are difficult to access. Assistance Dogs International (ADI), the organization that accredits service dog organizations in the United States, provides annual statistic on service dog teams from their accredited organizations [4]. ADI identifies competencies and sets the standards for the accreditation of service dog organizations. The ADA requires that the service dog be trained to perform one specific disability-related task for their handler, while ADI has set their requirement at a minimum of three. The amount of time to educate a service dog from an ADI-accredited organization is typically a minimum of 15 months. The cost of breeding, raising, training, and educating the individual who will be teamed with the service dog on how to work together, and ongoing lifetime support for the ADI-accredited service dog team can cost up to USD 50,000. ShareAmerica.com reported in 2016 that there were 500,000 service dog teams, including those who were self-taught and ADI-accredited teams, in the US, but this is an unsubstantiated reference [5]. In 2022, ADI reported that in the US, there were 14,809 assistance dog teams, of which, Service Dogs accounted for 7911, or 51% [4,5]. While this statistic does not include service dogs trained by their disabled handler with or without the assistance of a dog trainer, it is unlikely that this number would make up the difference reported by ShareAmerica.com.

The work of a service dog is focused on the person’s primary disability need, and those with disabilities may also have secondary needs [6]. Those teamed with a service dog may experience secondary benefits that further support their independence and ability to interact as fully as possible in society through a bond that develops with their service dogs. Humans have a strong emotional connection to canines, which elicits feelings of support and comfort and produces both psychological and physiological benefits [7,8,9,10,11]. Physiologic response to the human–canine bond include an increase in oxytocin, known as the bonding hormone, which is linked to positive emotional states. There is also an increase in dopamine and decrease in cortisol levels and blood pressure [12,13]. Psychological benefits of the human–canine bond include a range of responses, for example, research shows a decrease in depression [14,15,16,17], anxiety [14,16,17,18,19], and stress, [10,15,17]. The human–canine bond also benefits individuals in social connectedness [20,21,22,23,24,25,26,27,28], participation in activities of daily living [26,29,30,31,32], employment ability [22,28,33,34,35,36], and quality of life [11,34,37,38,39]. Additionally, being teamed with a service dog may benefit the individual through a reduction in prescribed medications, and/or a decrease in paid caregiver hours [29,39,40,41]. 

Benefits identified may support the value of service dogs for individuals who are teamed with service dogs. Yet, a review of the literature shows that the studies to date report on small sample sizes, are focused on individuals with a specific type of disability, such as PTSD, severe ambulatory diagnoses, or autism spectrum disorder, and/or may be specific to one group such as veterans. Service dogs work with individuals across a wide range of disabilities. Gaining understanding and generating evidence on the benefits of a broader group of individuals with disabilities is necessary to determine if the benefits identified in the limited studies to date are reported beyond individuals with those disease/ disability categories, or the specific groups studied. There are over 61 million people in the United States living with disabilities, yet less than 1% of this group are benefiting from being teamed with a service dog. Not all individuals may desire this type of support, and of those who do, some may not meet the ADA requirements. This area deserves to be explored to gain a greater understanding of the characteristics of those teamed with service dogs and the benefits they report. This evidence is essential to prevent this support from being a missed opportunity in the care of these individuals.

## 2. Materials and Methods [42]

This research is part of a larger study that also sought to better understand the values, assumptions, and beliefs about being teamed with service dogs, benefits of being teamed with service dogs, and experiences of receiving healthcare from the perspective of those teamed with a service dog. This research reports on the benefits of being teamed with service dogs.

### 2.1. Study Design

A cross-sectional survey design was utilized for the study. 

### 2.2. Participants

Participants were adults ≥18 years of age who identify as having visible and/or invisible disabilities and are teamed with a service dog.

### 2.3. IRB

The study was approved by the (Pace University IRB# 0004707). The survey identified that the person was being asked to help increase the understanding of the needs of individuals who are teamed with service dogs. Respondents were informed that the survey was anonymous and no personal information was collected. Signatures were not required. Survey recipients consented to completing the survey; once they clicked on the survey, it stated, prior to the questions: “By completing the following questions, I agree to participate in this survey study”.

### 2.4. Development and Pre-Testing

The Singleton Service Dog Survey (SSDS) was developed for this study to gain greater understanding of characteristics of individuals teamed with service dogs, and to describe: 1. service dog teams as a cultural community; 2. benefits of being teamed with a service dog for individuals across a wide range of disabilities; and 3. knowledge and cultural competence of healthcare providers for individuals with disabilities teamed with a service dog receiving care in healthcare settings. The tool was developed, and face validity was established from a review of the literature on these three areas of interest and with the input and review of a panel of experts in the field, including individuals teamed with service dogs. The survey layout/design, usability, and functionality were pre-tested with the review panel. This article reports for the first time on the SSDS Total, and on the benefits of being teamed with a service dog as measured using the SSDS Benefits Subscale. 

The SSDS comprises 25 questions: eight pertain to demographic characteristics to describe the sample and includes two fill-in-the-blanks for age and service dog tasks, while 17 questions utilize a 4-point scale (Table 1). The SSDS Total had a Cronbach’s alpha coefficient with a 95% confidence interval of 0.82 (Table 2). Three survey questions are related to the cultural community of service dog teams (SSDS Sub-Culture), seven survey questions identify benefits of being teamed with a service dog (SSDS Sub-Benefits), and seven survey questions relate to the knowledge of health care providers in the care individuals teamed with service dogs receive (SSDS Sub-Knowledge). The SSDS Benefits Subscale had a Cronbach’s alpha coefficient, with a 95% confidence interval, of 0.70 (Table 3).

### 2.5. Recruitment

The survey was created in Qualtrics, and data were collected using an open survey approach. Volunteers were sought for this study and were recruited in the United States through three organizations. Qualtrics targeted potential participants in their database who met the inclusion criteria, and two service dog organizations, Educated Canines Assisting with Disabilities and Palmetto Animal Assisted Life Services, sent the survey to clients in their databases. No incentives were offered by the researcher to participate. There was no randomization or adaptive questioning of the 32 items that were responded to over five screens. Respondents had the option of providing any additional information regarding being teamed with a service dog in a text box at the end of the survey. The survey was conducted from December 2021 through to March 2022. Manual checks for completeness were performed in the data analysis phase. IP addresses were checked to prevent multiple entries. View and participation rates were not calculated.

### 2.6. Analysis

Only completed questionnaires were analyzed. No time frame was specified, and no statistical corrections performed. Descriptive statistics, frequency, and percentages were calculated for the sample on all questions. 

## 3. Results

Two hundred and seventy individuals responded to the survey. A screening question on the primary benefit which the service dog supports resulted in identifying and screening out individuals who are teamed with assistance dogs for vision and hearing. Two hundred and four individuals teamed with a service dog met the inclusion criteria for the study. Survey respondents (*N* = 204) reported the primary benefit which the service dog supports as: mental/psychiatric (45%), or alert for medical condition (34%), or mobility (19%). Respondents had an average age of 38.87 years (SD = 13.47, SEM = 0.95, Min = 14.00, Max = 77.00, Skewness = 0.53, Kurtosis = −0.43) and were predominantly white (*n* = 156, 76.47%), non-Hispanic or Latino (*n* = 166, 81.37%), and Female (*n* = 114, 55.88%). Disability type was most frequently identified as physical and mental/ psychiatric (*n* = 79, 38.73%), followed by mental/ psychiatric (33%), and physical (28%). The majority of respondents were teamed with their service dogs for one to three years (*n* = 74, 36.27%). Training of the service dog by an Assistance Dog International-accredited service dog organization was reported by the majority of respondents (*n* = 113, 55.39%). Please see Table 4 for summary statistics.

Summary responses to the “benefits” questions are reported as frequency/percentages (Table 5). The range of responses across the first five of the seven benefits questions on emotional connections, community participation, and physical activity showed a high level of agreement of either somewhat or strongly agree combined responses (95–98%). The last two questions’ results showed a lower amount of somewhat or strongly agree combined responses, with benefits related to medications being the lowest (77%), followed by a decrease in paid care assistance hours (84%).

## 4. Discussion

This is the first study to examine benefits of being teamed with a service dog from the perspectives of individuals who are teamed with service dogs. These individuals have self-selected this type of support, and as such, the findings help to identify their perspectives on ways in which they benefit from a service dog. It is not surprising that these individuals report benefits. However, the strength of their agreement to the benefits identified can be influenced by many factors. In this study, strongly positive responses to the benefits of service dogs were reported.

Clarifying and screening for the assistance dog category of service dogs helps us to be more specific and accurate about benefits reported for this group. To the best of this author’s knowledge, this study is the largest sample to date of individuals in the United States teamed with service dogs as defined by ADI. As such, it offers many critical insights into the characteristics of individuals teamed with service dogs and the benefits these individuals report. 

This study adds empirical evidence that supports benefits of being teamed with a service dog as previously reported in limited studies and anecdotal information in the literature. Additionally, this study substantiates that respondents overwhelmingly reported benefits from being teamed with a service dog that went beyond their primary needs. Benefits identified on the SSDS include social and emotional connectedness, physical activity, psychological wellbeing, quality of life, a decrease in prescribed medications, and a reduction in paid care hours. Research to date on the benefits identified in the SSDS have been with small samples, and/or focused on one type of disability, and/or on other types of assistance dogs for vision and hearing support [11,20,21,22,23,24,25,26,27,28,29,32,33,34,35,36,39,41].

Gaining an understanding of the economics related to service dogs is an important category of benefits to consider. Respondents reported that being teamed with a service dog may help to decrease medications, which may have both an economic and health benefit. Additionally, respondents reported that those teamed with a service dog may have a reduction in care hours, which can have an economic benefit for paid care hours. Past studies that have reported on these two areas are dated and report on specific groups such as those with ambulatory disabilities [29,39,41]. This present study with individuals with a range of disabilities reports positive benefits overall in both areas. However, general, non-specific questions asked in this study do not provide the level of detail to better understand and define these benefits. These types of economic benefits should be explored and quantified in future studies to identify if any gender, race, or ethnic group benefits more, and the role that time and/or changes in disability over time may have for these economic benefits. 

Within this sample, an interesting finding is that respondents were predominantly female, white, and non-Hispanic/Latino. More research is needed with larger samples to better understand if this finding is specific to this sample or is occurring in the population of individuals teamed with service dogs. This may indicate a need for greater diversity, equity, and inclusion to access, availability, resources, and support for all those with disabilities who desire and are suitable to be teamed with service dogs. Presently, less than 1% of the 61 million people in the United States living with disabilities (the largest minority group in the United States today) are teamed with a service dog. Acknowledging that not all individuals may desire to be teamed with a service dog, and of those who do many may not meet the ADA requirements, there is great inequity seen in the number of those who may benefit versus the number of those presently benefiting from being teamed with a service dog. Given the number of people with disabilities, and the very small percentages of these individuals who are teamed with service dogs, it becomes evident that the support from service dogs is a missed opportunity for some. 

This study provides a snapshot of individuals who have chosen to be teamed with a service dog using a wider lens to capture a larger sample. Being teamed with a service dog is initially focused on supporting the primary need of the individual’s disability. The relationship or bond between the handler and service dog emerges, evolves, grows, and changes over time. Choosing to be teamed with a service dog reflects self-determination in a therapeutic approach to support independence for individuals living with disabilities and follows the philosophy, principles, and practice of canines assisting in health.

Limitations of this study include a non-random sample with no denominator available. This limits the generalizability of the study results and should be considered in future research with individuals teamed with service dogs. Important considerations for future research in this area should also include barriers to a person being teamed with a service dog. This must be explored at all levels, including the individual, organizational, and service levels. Additionally, future research to gain a better understanding of the health outcome benefits for those teamed with service dogs as compared to those who are not teamed with service dogs will support evidence-based practice for individuals with disabilities. 

## 5. Conclusions

Service dogs are broadly helpful across all ages, genders, races, and ethnicities, and are equally beneficial for individuals living with mental–psychiatric, physical, and mental–psychiatric/physical disabilities. Given the history of service dogs and the benefits of those teamed with service dogs reported in this study, it is incumbent upon society to support its social contract with individuals with disabilities by making this proven support more readily available. This will require a greater education of healthcare professionals to be knowledgeable about service dogs in order to assist patients and direct them to resources. Additionally, healthcare professionals will need to gain knowledge and cultural competence in caring for individuals teamed with service dogs. With increased knowledge and competence in caring for individuals teamed with service dogs, healthcare professionals may wish to consider recommending a service dog as part of a plan of care, when appropriate, to support independence for patients living with visible and invisible disabilities.

## Figures and Tables

**Table 1:** Singleton Service Dog Survey.

Screening question:	
What is the primary support your service dog provides	_______________
1. What is your age? __________	
2. What is your race? __________	
a.	American Indian or Alaska Native
b.	Asian
c.	Black or African American
d.	Native Hawaiian or other Pacific Islander
e.	White
f.	Multi-racial
g.	Unknown
3. What is your ethnicity?	
a.	Hispanic or Latino
b.	Non-Hispanic or Latino
4. What is your gender/ How do you identify?	
a.	Male
b.	Non-binary
c.	Female
d.	Prefer not to answer
5. My disabilities are	
a.	Physical
b.	Mental
c.	Physical and mental
6. How long have you been teamed with as service dog?	
a.	Less than 1 year
b.	1–3 years
c.	3–5 years
d.	5–7 years
e.	7+ years
7. How was your service dog trained?	
a.	Accredited Organization: Assistance Dogs International
b.	Self-taught with dog trainer
c.	Self-taught
8. What three tasks do your service dog perform to mitigate your disability?	
1.____________________2.____________________3.____________________	
Response choices questions 9–25: Strongly Agree (4), Slightly Agree (3), Slightly Disagree (2), Strongly Disagree (1)	
9. Dogs can be trained to assist humans	
10. Service dogs can lessen disabilities	
11. I value the task(s) that service dogs perform to help individuals with visible and invisible disabilities	
12. My service dog helps to improve my emotional connections to others	
13. My service dog helps me to participate in my community	
14. My serviced dog helps me to be physically active	
15. My service dog helps me with my psychological wellbeing	
16. My service dog helps me to have a good quality of life	
17. Having a service dog has helped my health care provider to decrease my mediations	
18. Having a service dog has helped to decrease the assistance hours, paid and/or unpaid hours, for care I receive to support my needs	
19. Healthcare providers recognize me as the expert in working with a service dog and communicate with me about how to best meet our needs when receiving care	
20. Healthcare providers know that my service dog and I are a team, my service dog mitigates my disability and lets me be independent and interact in society	
21. Healthcare providers make me, not my service dog, the focus when I am receiving care	
22. Healthcare providers know that it is okay to acknowledge my service dog, to me, but not to interact with my service dog without my directing the interaction	
23. Healthcare providers are aware of my legal protections through the Americans with Disabilities Act	
24. Healthcare providers are aware of their healthcare organization’s policy on assistance animals	
25. Healthcare providers advocate for me and my service dog should this be needed	

**Table 2:** Reliability table for SSDS Total.

Scale	No. of Items	α	Lower Bound	Upper Bound
SSD: All	17	0.84	0.82	0.86

**Table 3:** Reliability table for SSDS Sub2: Benefits.

Scale	No. of Items	α	Lower Bound	Upper Bound
Sub2: Benefits	7	0.70	0.66	0.75

**Table 4:** Frequency table for nominal and ordinal variables.

Variable	*n*	*%*
Race		
American Indian or Alaska Native	3	1.47
Asian	7	3.43
Black or African American	24	11.76
Multi-racial	11	5.39
Native Hawaiian or other Pacific Islander	1	0.49
Unknown	2	0.98
White	156	76.47
Missing	0	0.00
Gender		
Female	114	55.88
Male	83	40.69
Non-binary/Third gender	7	3.43
Missing	0	0.00
Disability		
Mental	68	33.33
Physical and Mental	79	38.73
Physical	57	27.94
Missing	0	0.00
Time-teamed		
Less than 1 year	32	15.69
1–3 years	74	36.27
3–5 years	55	26.96
5–7 years	18	8.82
7+ years	25	12.25
Missing	0	0.00
Training		
Self-taught with Dog Trainer	47	23.04
Self-taught	44	21.57
Accredited Service Dog: ADI	113	55.39
Missing	0	0.00

Note. Due to rounding errors, percentages may not equal 100%.

**Table 5:** Benefits of being teamed with a service dog.

Question *	Strongly	Somewhat	Somewhat	Strongly
	Agree	Agree	Disagree	Disagree
1. My service dog helps to improve my emotional connections to others	79%	17%	2%	1%
2. My service dog helps me to participate in my community	73%	24%	2%	1%
3. My serviced dog helps me to be physically active	75%	20%	2%	1%
4. My service dog helps me with my psychological wellbeing	85%	13%	2%	0.49%
5. My service dog helps me to have a good quality of life	84%	13%	1%	6%
6. Having a service dog has helped my health care provider to decrease my mediations	48%	31%	15%	6%
7. Having a service dog has helped to decrease the assistance hours, paid and/or unpaid hours, for care I receive to support my needs	54%	30%	9%	6%

* Across all 7 questions, there was a 1% no response rate.

## Data Availability

The data presented in this study are available upon request from the corresponding author. The data are not publicly available due to privacy and ethical reasons.
